# Association between weight-adjusted waist index and chronic obstructive pulmonary disease

**DOI:** 10.1371/journal.pone.0334922

**Published:** 2025-10-22

**Authors:** Xingshi Hua, Yu Gan, Xiaodong Lv

**Affiliations:** 1 Liaoning University of Traditional Chinese Medicine, Shenyang, China; 2 The Second Affiliated Hospital of Liaoning University of Traditional Chinese Medicine, Shenyang, China; Hamadan University of Medical Sciences, School of Public Health, IRAN, ISLAMIC REPUBLIC OF

## Abstract

**Objective:**

This study aimed to investigate the association between the weight-adjusted waist index (WWI), a novel obesity metric, and the prevalence of chronic obstructive pulmonary disease (COPD) in a nationally representative sample of U.S. adults, and to compare its predictive utility for COPD against conventional obesity indices.

**Methods:**

This cross-sectional study utilized data from the 2017–2020 National Health and Nutrition Examination Survey (NHANES). COPD diagnosis was based on self-report. The association between WWI and COPD was investigated using multivariable logistic regression models, adjusting for key covariates including age, gender, race/ethnicity, smoking status, hypertension, and diabetes. Restricted cubic splines (RCS) were used to explore potential non-linear relationships. Receiver operating characteristic (ROC) curves were used to assess WWI’s predictive performance. All statistical analyses were conducted using R software, accounting for the complex survey design and weighting.

**Results:**

This study comprised 3,111 participants, among whom the prevalence of COPD was 8.5%. The findings indicated a significant positive association between WWI and the prevalence of COPD (OR = 1.30, 95% CI: 1.02–1.66). When analyzed by quartiles, a significant positive dose-response relationship was observed (*P* for trend = 0.031). Furthermore, receiver operating characteristic (ROC) analysis revealed that WWI had significantly better predictive performance for COPD (Area Under the Curve [AUC] = 0.662) than conventional obesity indices.

**Conclusion:**

Our findings suggest a significant positive association between WWI and the self-reported prevalence of COPD. WWI shows promise as a simple, non-invasive anthropometric tool that may aid in identifying individuals with higher odds of having COPD in clinical and public health settings.

## Introduction

Chronic obstructive pulmonary disease (COPD) is a highly prevalent condition that is both preventable and treatable [1]. It is primarily characterized by persistent airflow obstruction and respiratory symptoms, which commonly result from prolonged exposure to harmful particles or gases [2]. Prolonged exposure leads to significant structural changes in the airways and lung parenchyma [3]. Common symptoms of COPD include breathlessness, a persistent cough, and increased mucus production [4]. Furthermore, many patients with COPD have comorbidities that increase disability and mortality [5]. COPD represents a major global health challenge and, according to the latest Global Burden of Disease (GBD) 2021 study, it remains one of the leading causes of death worldwide [6].

Among the various modifiable risk factors for COPD, obesity has emerged as a significant contributor to its pathophysiology and burden [7]. However, standard anthropometric measures like Body Mass Index (BMI) have notable limitations. BMI lacks the ability to separate fat from muscle mass, does not effectively capture the distribution of adipose tissue, and its applicability may vary due to differences in race and gender [8–12]. To overcome these limitations, the weight-adjusted waist index (WWI) has been introduced as a more accurate metric for assessing central obesity [13,14]. The WWI is derived by taking the ratio of waist circumference to the square root of body weight, offering a more precise estimation of abdominal fat accumulation [15,16].

The utility of WWI is supported by evidence demonstrating its strong association with increased abdominal fat and its greater stability across diverse populations [17,18]. Furthermore, WWI has exhibited more robust connections with metabolic abnormalities, such as insulin resistance and proteinuria, in comparison to BMI, suggesting it may act as a more effective marker for assessing the risk of obesity-related metabolic disorders [19,20]. These obesity-driven pathologies, particularly the chronic low-grade inflammation and mechanical lung compression associated with central adiposity, offer a direct mechanistic pathway to the development and progression of COPD [21]. It is important to note that, unlike BMI, WWI does not currently have universally established cut-off values for diagnosing obesity. While some studies have proposed population- and outcome-specific thresholds [22], no universally accepted cut-offs exist. Given these specificities and the multi-ethnic nature of the National Health and Nutrition Examination Survey (NHANES) population, our study analyzes WWI as a continuous variable to evaluate its associated health risks.

While the link between general obesity and COPD is known, the specific association between the WWI and COPD, particularly within a large and diverse national population, has not been thoroughly investigated. Therefore, this study aims to address this gap by investigating the association between WWI and the prevalence of COPD, characterizing its dose-response relationship, and comparing its predictive utility against conventional anthropometric indices, using data from the 2017–2020 cycles of NHANES, encompassing a final sample of 3,111 adult participants.

## Methods

### Study design and sample

This study utilized data from the 2017–2020 NHANES, accessed by the authors starting in December 2024, to conduct a cross-sectional investigation aimed at exploring the potential association between the WWI and COPD. NHANES is a comprehensive survey conducted across the United States to evaluate the health and nutritional status of individuals living outside of institutional settings. It provides comprehensive data on health conditions, dietary intake, and demographic characteristics. NHANES employs an advanced, stratified, and multistage sampling approach, ensuring that the chosen participants accurately represent the broader population of the United States, thus enabling the creation of nationally representative health statistics. Comprehensive details on NHANES study methodology, data, and codebooks are publicly accessible on the official website: https://www.cdc.gov/nchs/nhanes/. This analysis used the publicly available, de-identified data files, and the authors did not have access to participants’ personally identifiable information.

**Fig 1:**
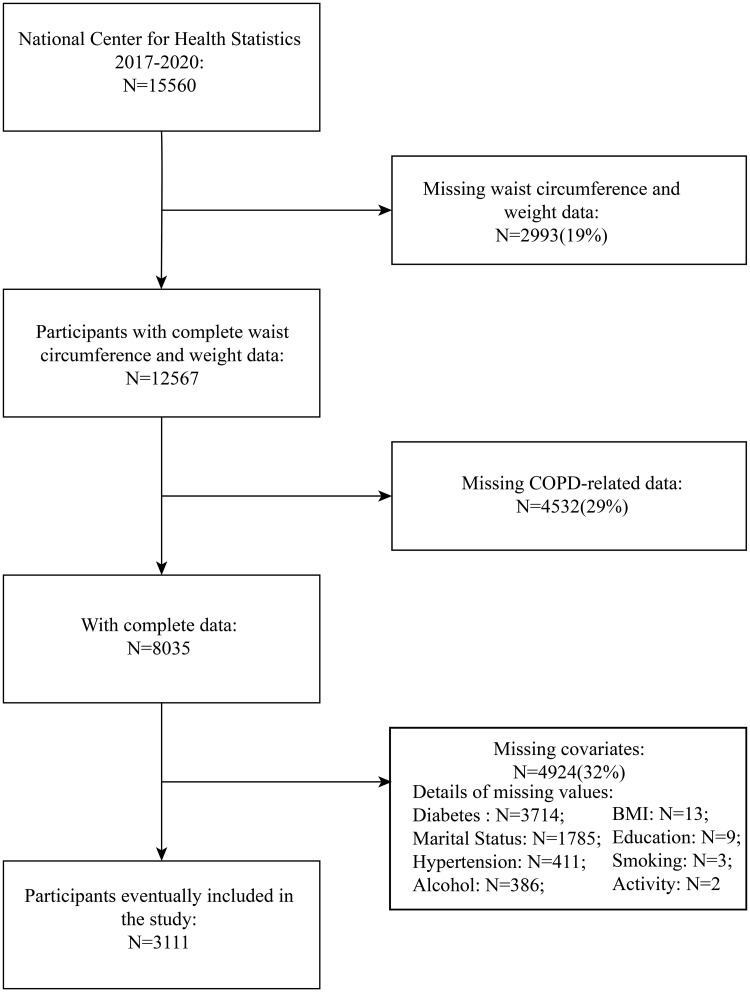
Flowchart of participant selection from the National Health and Nutrition Examination Survey (NHANES) 2017–2020. Abbreviations: BMI, body mass index; COPD, chronic obstructive pulmonary disease. Total excluded represents the number of unique participants with missing data in at least one variable. The sum of missing values for each variable is greater than the total excluded due to overlapping missingness.

The study protocol for NHANES was approved by the National Center for Health Statistics (NCHS) Research Ethics Review Board, and all participants provided written informed consent prior to data collection. This study utilized these publicly available, de-identified data for secondary analysis. The initial sample included 15,560 individuals who contributed valid data to the NHANES survey conducted between 2017 and 2020. To maintain data accuracy and uphold the rigor of the study, individuals with missing key health indicators (such as waist circumference, body weight, and COPD diagnosis data) or incomplete covariate information were excluded. The specific process for selecting the sample and the criteria for exclusion are comprehensively outlined in Fig 1. After applying these exclusion criteria, a final analytical sample of 3,111 participants was established. This study employed a complete case analysis, meaning only participants with complete data for all included variables were retained.

Additionally, data processing and analysis in this study adhered to the NHANES statistical guidelines, incorporating weighting measures to address biases introduced by the sampling design. These measures helped to ensure the inferential validity and statistical reliability of the results, enabling the study’s conclusions to more precisely portray the overall health status and trends of the U.S. population.

### Exposure variable: WWI

Within this analysis, WWI was the main exposure variable, determined by calculating the ratio of waist circumference (cm) to the square root of body weight (kg). Data on waist circumference and body weight were sourced from the “Body Measurements” section within the NHANES examination dataset. To ensure measurement accuracy, all measurements were conducted by professionally trained healthcare personnel following standardized procedures. During weight measurement, participants wore examination clothing, stood barefoot, and maintained a relaxed posture to ensure consistency and precision. Following the official NHANES protocol, waist circumference was measured at the uppermost lateral border of the right iliac crest, with the tape positioned horizontally around the abdomen. Body weight was measured to the nearest 0.1 kg using a calibrated digital scale.

To thoroughly examine its link with obesity levels and related health risks, WWI was first treated as a continuous variable and then divided into four quartiles (Q1-Q4).

### Outcome variable: COPD

The outcome variable in this study was the presence of COPD, which was determined through self-report. Data were sourced from the NHANES “Medical Conditions” questionnaire file. Participants were classified as having COPD if they answered “yes” to the question: “Ever told you had COPD, emphysema, Chronic Bronchitis (ChB)?”. While post-bronchodilator spirometry is the gold standard for clinical diagnosis, self-reported diagnoses are a common and accepted method for assessing disease prevalence in large-scale epidemiological surveys like NHANES due to their feasibility and have demonstrated reasonable validity [23]. It is important to acknowledge, however, that this approach is inherently prone to misclassification, as participants may confuse conditions like acute and chronic bronchitis, and is also subject to recall bias [24,25]. Such non-differential misclassification would likely attenuate the true association, biasing our results toward the null [26].

### Covariates

Based on a review of previous literature and their clinical relevance, we selected a range of potential confounders [27–30]. Demographic data included age (continuous), gender (male or female), and race/ethnicity (categorized as Mexican American, Non-Hispanic White, Non-Hispanic Black, or Other Race). Socioeconomic and lifestyle factors included education level (grouped as Below High School or High School and Above), marital status (Married/Living with partner or Single), smoking status (current smoker or not), alcohol consumption (current drinker or not), and physical activity. Physical activity was dichotomized based on participants’ self-report. Individuals were classified as ‘active’ if they reported engaging in at least 10 continuous minutes of either moderate- or vigorous-intensity recreational activities in a typical week. Those who reported engaging in neither were classified as ‘inactive’.

Key comorbidities were also ascertained. Hypertension was defined as a self-reported physician diagnosis, an average systolic blood pressure ≥140 mmHg, or an average diastolic blood pressure ≥90 mmHg. Diabetes was defined by a self-reported physician diagnosis or a fasting plasma glucose level ≥7.0 mmol/L. The classification and coding of all categorical variables were performed in strict accordance with the official codebooks and guidelines provided in the NHANES documentation for the respective survey years.

### Statistical analyses

This study employed a detailed statistical approach to explore the relationship between WWI, considered as a continuous variable, and COPD, categorized as a binary variable. Participant characteristics were summarized with continuous variables presented as mean ± standard deviation (SD) and categorical variables as proportions. Participants were divided into two distinct groups based on their COPD diagnosis. To assess differences in continuous data between these groups, weighted t-tests were performed. For categorical data, weighted chi-square tests were used to evaluate the variations.

The association between WWI and COPD was assessed using three multivariable logistic regression models. Model I was the crude, unadjusted model. Model II was adjusted for core demographic variables: age, gender, and race/ethnicity. Model III, our fully adjusted model, was adjusted for several covariates. These variables were selected based on their established roles as major risk factors for COPD and important potential confounders as supported by existing literature, and included education level, marital status, smoking status, alcohol consumption, physical activity, hypertension, and diabetes. This hierarchical approach was chosen to systematically evaluate the association while incrementally controlling for different layers of confounding.

To further explore the dose-response relationship, restricted cubic splines (RCS) were fitted to the multivariable logistic regression model. This method allowed for a flexible examination of the potential non-linear association between continuous WWI and the odds of COPD, and the linearity assumption was assessed by testing the significance of the non-linear spline terms. To assess the possible moderating influences of various factors, subgroup analyses were performed, stratified by age, gender, race, education level, marital status, smoking status, physical activity, hypertension, and diabetes. The statistical significance of any effect modification was formally tested by incorporating an interaction term between WWI and each subgroup variable into the model. All statistical analyses were conducted using R software (version 4.4.1), taking into account the complex sampling design and weighting applied in NHANES data. The ‘survey’, ‘rms’, and ‘pROC’ packages were used for these analyses. Statistical significance was determined at a threshold of **P* *< 0.05.

## Results

### Baseline characteristics of participants

The baseline characteristics of the 3,111 participants included in the final analysis are presented in Table 1. The overall prevalence of COPD was 8.5% (N = 263). Compared with participants without COPD, those with the condition were significantly older. The proportion of Non-Hispanic White individuals was higher in the COPD group, which was also characterized by a greater prevalence of current smokers and physical inactivity. Similarly, the prevalence of both hypertension and diabetes was significantly higher among participants with COPD (*P* < 0.001 for both). Furthermore, participants with COPD had significantly higher mean values for WWI, BMI, and WC.

**Table 1:** Baseline Characteristics of Participants in the NHANES 2017–2020, Categorized by COPD Status.

Characteristic	Overall N = 3111	COPD N = 263	Non-COPD N = 2848	*P*-value
**WWI**	11.01 ± 0.85	11.46 ± 0.79	10.96 ± 0.84	<0.001
**Age (years)**				<0.001
<40	1,006 (38%)	29 (13%)	977 (41%)	
40-64	1,412 (43%)	135 (57%)	1,277 (42%)	
>=65	693 (18%)	99 (30%)	594 (17%)	
**Gender**				0.237
Female	1,438 (47%)	115 (52%)	1,323 (46%)	
Male	1,673 (53%)	148 (48%)	1,525 (54%)	
**Race**				<0.001
Mexican American	402 (9%)	12 (3%)	390 (9%)	
Non-Hispanic Black	768 (10%)	52 (7%)	716 (11%)	
Non-Hispanic White	1,056 (64%)	150 (79%)	906 (62%)	
Other Race	885 (17%)	49 (11%)	836 (18%)	
**Education Level**				0.253
Below High School Level	531 (10%)	54 (12%)	477 (9%)	
High School Level and Above	2,580 (90%)	209 (88%)	2,371 (91%)	
**Marital Status**				0.229
Married/Living with partner	2,381 (78%)	206 (82%)	2,175 (78%)	
Single	730 (22%)	57 (18%)	673 (22%)	
**Smoking Status**				<0.001
No	1,812 (57%)	63 (21%)	1,749 (61%)	
Yes	1,299 (43%)	200 (79%)	1,099 (39%)	
**Physical Activity**				<0.001
Active	1,513 (56%)	70 (35%)	1,443 (58%)	
Inactive	1,598 (44%)	193 (65%)	1,405 (42%)	
**Alcohol Consumption**				0.052
No	279 (6%)	14 (3%)	265 (7%)	
Yes	2,832 (94%)	249 (97%)	2,583 (93%)	
**Hypertension**				<0.001
No	1,672 (59%)	95 (43%)	1,577 (61%)	
Yes	1,439 (41%)	168 (57%)	1,271 (39%)	
**Diabetes**				<0.001
No	2,202 (77%)	141 (58%)	2,061 (79%)	
Yes	909 (23%)	122 (42%)	787 (21%)	
**BMI (kg/m²)**	30.04 ± 7.29	32.08 ± 9.12	29.85 ± 7.06	0.018
**WC (cm)**	101.60 ± 17.61	107.90 ± 19.15	101.00 ± 17.34	<0.001
**Weight (kg)**	86.05 ± 22.84	90.15 ± 27.07	85.66 ± 22.37	0.113

Data are presented as mean *±* *standard deviation* (*SD*) for continuous variables or unweighted n (weighted %) for categorical variables. P-values were derived from weighted independent t-tests for continuous variables and weighted chi-square tests for categorical variables to account for the complex survey design.

Abbreviations: BMI, body mass index; COPD, chronic obstructive pulmonary disease; WC, waist circumference; WWI, weight-adjusted waist index.

### Association between WWI and COPD

After full adjustment in Model III, WWI remained significantly associated with COPD (Table 2). When analyzed as a continuous variable, each unit increase in WWI was associated with a 30% increase in the odds of having COPD (OR = 1.30, 95% CI: 1.02–1.66, *P* = 0.034). In the quartile analysis, participants in the highest WWI quartile (Q4) had an 86% increase in the odds of COPD compared to those in the lowest quartile (Q1) (OR = 1.86, 95% CI: 1.11–3.11, *P* = 0.022), and the test for trend across quartiles was significant (*P* for trend = 0.031).

**Table 2:** Multivariable logistic regression models for the association between WWI and COPD.

	Model I (Unadjusted)		Model II (Demographics Adjusted)		Model III (Fully Adjusted)	
Variable	OR (95% CI)	*P*-value	OR (95% CI)	*P*-value	OR (95% CI)	*P*-value
WWI Continuous	2.02 (1.65, 2.47)	<0.001	1.54 (1.20, 1.96)	0.002	1.30 (1.02, 1.66)	0.034
Categories						
Quartile1	Ref		Ref		Ref	
Quartile2	2.60 (1.27, 5.32)	0.011	1.88 (0.87, 4.09)	0.10	1.54 (0.72, 3.31)	0.2
Quartile3	4.03 (1.79, 9.07)	0.002	2.61 (1.13, 5.99)	0.027	1.75 (0.87, 3.51)	0.11
Quartile4	5.85 (3.11, 11.0)	<0.001	3.00 (1.53, 5.85)	0.003	1.86 (1.11, 3.11)	0.022
*P* for trend	3.61 (2.23, 5.85)	<0.001	2.25 (1.36, 3.70)	0.003	1.56 (1.05, 2.31)	0.031

Abbreviations: OR, odds ratio; CI, confidence interval; WWI, weight-adjusted waist index; Ref, reference category.

Model I: Unadjusted.

Model II: Adjusted for age, gender, and race/ethnicity.

Model III: Adjusted for age, gender, race/ethnicity, education level, marital status, smoking status, physical activity, alcohol consumption, hypertension, and diabetes.

P for trend was derived by modeling the WWI quartiles as an ordinal variable to test for a linear trend across the groups. The corresponding OR represents the increase in odds for each one-quartile increase in WWI.

### Dose-response relationship analysis

**Fig 2:**
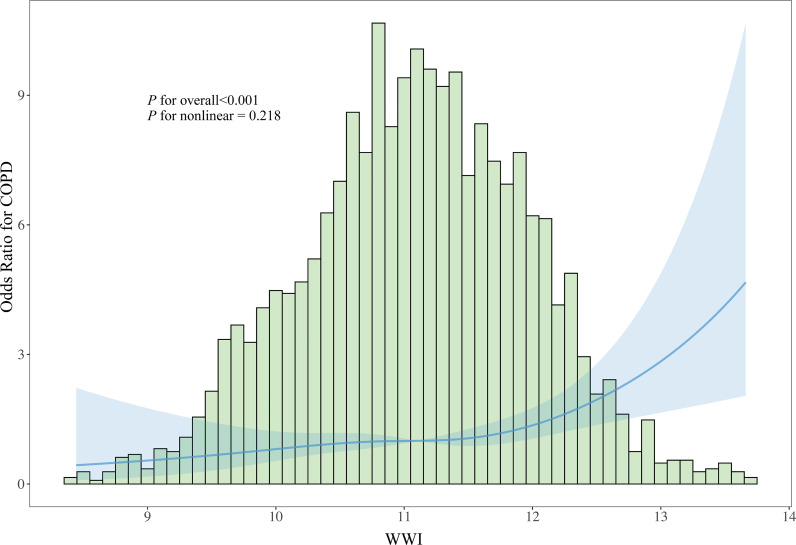
Dose-response relationship between WWI and the odds of COPD. The solid blue line represents the odds ratio with WWI as a continuous variable, and the shaded area indicates the 95% confidence intervals. The histogram displays the population distribution of WWI values. The analysis was adjusted for age, gender, race/ethnicity, education level, marital status, smoking status, physical activity, alcohol consumption, hypertension, and diabetes.

To further explore the dose-response relationship between WWI and COPD, we performed RCS analysis based on the fully adjusted Model III. The RCS analysis revealed a significant overall association between WWI and the odds of having COPD (*P* for overall < 0.001), while the test for non-linearity was not statistically significant (*P* for non-linearity = 0.218), indicating a predominantly linear relationship between WWI and the odds of COPD across the observed range of WWI values (Fig 2). The curve demonstrates a consistent positive association, with the odds ratio for COPD steadily increasing as WWI values rise.

### Subgroup analysis

**Fig 3:**
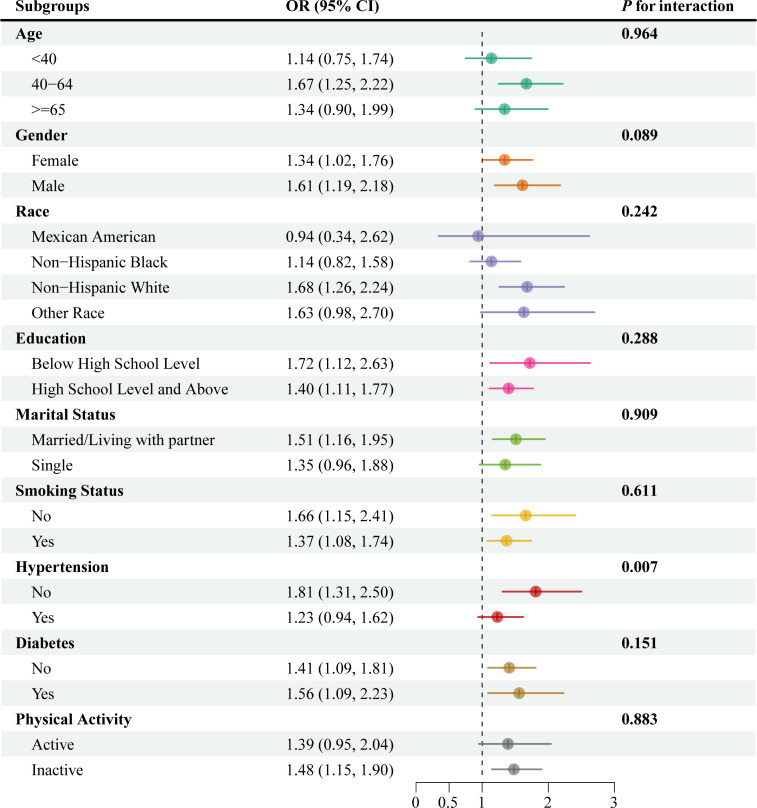
Subgroup analysis demonstrating the association between WWI and the odds of COPD across various demographic and clinical characteristics. All analyses were based on the fully adjusted Model III. The consistent direction of the association across most subgroups supports the robustness of the main findings. OR, odds ratio; CI, confidence interval.

To assess the robustness of our findings, we conducted subgroup analyses across various demographic and clinical characteristics, which also served as sensitivity analyses (Fig 3). The positive direction of the association between WWI and the odds of COPD was largely consistent across the majority of subgroups, supporting the stability of our main findings. Although statistical significance was not reached in some smaller subgroups, likely due to reduced statistical power, the overall consistency reinforces that the relationship between WWI and COPD is robust across diverse population characteristics.

Interaction testing revealed that only hypertension status significantly modified this association (*P* for interaction = 0.007). Specifically, the association was stronger and statistically significant among participants without hypertension (OR = 1.81, 95% CI: 1.31–2.50) compared to those with hypertension (OR = 1.23, 95% CI: 0.94–1.62). No significant interactions were detected for the other variables (all *P* for interaction > 0.05), further supporting the general consistency of the WWI-COPD association.

### Model performance evaluation

**Fig 4:**
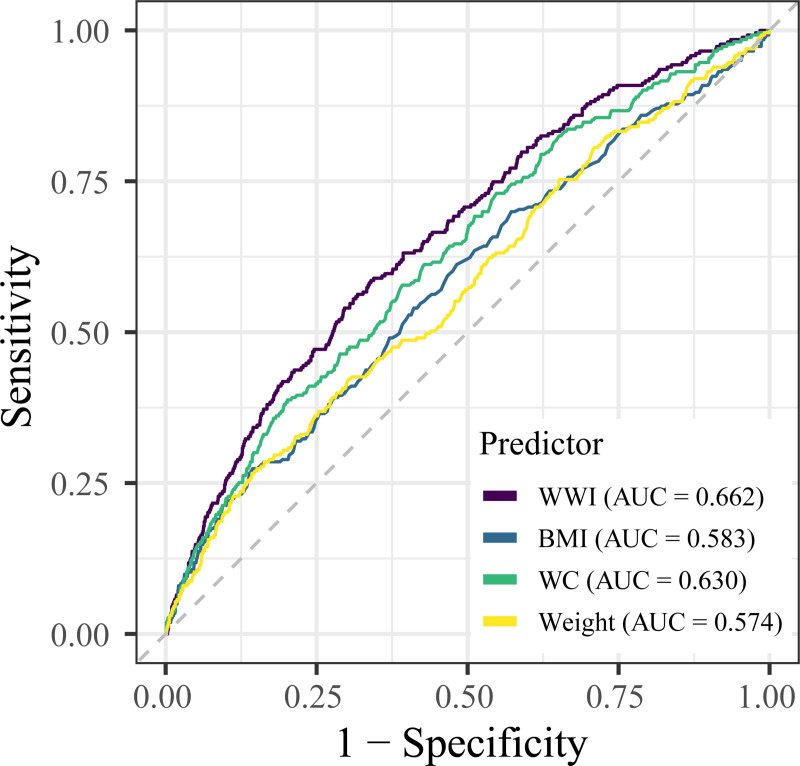
ROC curves comparing the predictive performance of different obesity indices for COPD. Abbreviations: AUC, Area Under the Curve; BMI, body mass index; COPD, chronic obstructive pulmonary disease; ROC, Receiver Operating Characteristic; WC, waist circumference; WWI, weight-adjusted waist index.

To compare the predictive value of WWI with traditional anthropometric indices for COPD, we performed ROC curve analyses (Fig 4). As detailed in Table 3, all four obesity indicators demonstrated some predictive ability, with WWI yielding the largest Area Under the Curve (AUC) of 0.662 (95% CI: 0.628–0.697). To formally test for statistical differences, we used the DeLong test to compare the AUC of WWI against the other indices. The results showed that WWI had a higher AUC compared to the other indices, and the DeLong test confirmed these differences were statistically significant for BMI (*P* < 0.001), WC (*P* = 0.035), and Weight (*P* < 0.001). These findings suggest that WWI has a superior predictive performance for identifying COPD compared to other commonly used obesity measures in this population.

**Table 3:** Predictive performance of different obesity indices for COPD.

Test	AUC	95% CI	*P*-value (vs. WWI)	Best threshold	Specificity	Sensitivity
WWI	0.662	0.628–0.697	—	11.42	0.660	0.586
BMI	0.583	0.546–0.621	<0.001	29.05	0.518	0.612
WC	0.630	0.594–0.665	0.035	104.65	0.608	0.578
Weight	0.574	0.536–0.611	<0.001	107.15	0.857	0.266

Abbreviations: AUC, Area Under the Curve; BMI, body mass index; CI, Confidence Interval; COPD, chronic obstructive pulmonary disease; WC, waist circumference; WWI, weight-adjusted waist index.

P-values were calculated using the DeLong test for comparing the AUC of each indicator against the AUC of WWI.

Furthermore, we calculated the Integrated Discrimination Improvement (IDI) and Net Reclassification Improvement (NRI) to assess the incremental value of the models. Both metrics confirmed a significant improvement in performance for the adjusted models compared to the unadjusted model (all *P* < 0.001; Table 4).

**Table 4:** Model performance evaluation of different models for COPD.

Comparison	IDI (95% CI)	*P*-value	NRI (95% CI)	*P*-value
**Model 2 vs. Model 1**	0.0275 (0.0184, 0.0405)	< 0.001	0.6202 (0.4651, 0.7240)	< 0.001
**Model 3 vs. Model 1**	0.1167 (0.0941, 0.1578)	< 0.001	0.9275 (0.8025, 1.0418)	< 0.001
**Model 3 vs. Model 2**	0.0892 (0.0694, 0.1244)	< 0.001	0.8098 (0.7361, 0.9644)	< 0.001

Model I: Unadjusted.

Model II: Adjusted for age, gender, and race/ethnicity.

Model III: Adjusted for age, gender, race/ethnicity, education level, marital status, smoking status, physical activity, alcohol consumption, hypertension, and diabetes.

CI, confidence interval; IDI, integrated discrimination improvement; NRI, net reclassification improvement.

## Discussion

In this study of 3,111 participants, a cross-sectional analysis revealed a statistically significant positive association between WWI and COPD. Further examination through stratified subgroup analyses indicated that this positive association was generally robust across most key demographic and clinical subgroups, suggesting a broad applicability of our main finding. This general consistency underscores the potential role of WWI as an independent and stable indicator for assessing the odds of having COPD, thereby highlighting the significant contribution of central obesity in the pathogenesis of the disease. These findings suggest that managing central obesity, as measured by WWI, may be an important component of public health strategies aimed at mitigating the burden of COPD.

A key finding of our study is that WWI is not only associated with COPD but also appears to be a superior predictor compared to traditional anthropometric measures. Two key findings from our analysis support this conclusion. First, the association between WWI and COPD remained robust even after full adjustment for a wide range of confounders, with each unit increase in WWI corresponding to a 30% increase in the odds of having COPD (OR = 1.30; 95% CI: 1.02–1.66). Second, our formal comparison of predictive models using ROC analysis demonstrated that WWI had a statistically superior predictive performance for identifying COPD compared to other common indices, with the most pronounced improvement seen over BMI and Weight (*P* < 0.001 for both). While traditional indices like BMI are valuable, they cannot distinguish between fat and muscle mass. WWI, by integrating both waist circumference and body weight, appears to better capture the specific obesity phenotype linked to COPD [31–33]. This enhanced, statistically validated performance suggests that WWI warrants further investigation as a potentially more informative clinical and epidemiological tool [15].

Further exploration of our findings revealed several nuances. The analysis of the dose-response relationship using restricted cubic splines confirmed a significant, positive, and broadly linear association between WWI and the odds of COPD (*P* for overall < 0.001; *P* for non-linear = 0.218). This linear trend suggests a consistent increase in the odds of having COPD with each increment in WWI. Additionally, the significant interaction we observed with hypertension (*P* for interaction = 0.007) suggests that the impact of central obesity on COPD prevalence may be modified by the presence of this comorbidity. This finding warrants further investigation to understand how these factors synergize and to potentially tailor preventive strategies for high-risk subgroups. As this was an exploratory finding, we can only hypothesize about the underlying mechanism. One possible explanation is that in patients with established hypertension, the underlying vascular and systemic inflammatory pathways are already highly active, potentially masking the additional, relative contribution of central obesity to COPD risk [34].

Previous research has extensively investigated the risk factors associated with COPD. For instance, Holtjer et al.‘s umbrella review, which encompassed 75 reviews, summarized 45 risk factors for COPD, including a high BMI [35]. Beijers et al. emphasized the critical role of dietary and nutritional strategies in both the prevention and management of COPD [36]. Furthermore, Brock et al. elucidated the notable mechanical effects of obesity on lung function and its potential link to pulmonary diseases, thus offering a plausible explanation for the association between obesity and lung diseases [37]. Another sizable prospective study conducted among the general Japanese population also demonstrated a significant correlation between BMI, changes in body weight, and mortality from COPD [38].

Research indicates that there is a significant association between obesity, as determined by BMI or WWI, and the severity of COPD, as well as its overall prognosis. Obesity is closely interconnected with metabolic syndrome, a condition that can intensify the pathophysiological mechanisms of COPD, particularly through inflammatory responses and oxidative stress [39–42]. The systemic inflammation found in individuals with obesity can further contribute to the decline in lung function, thus exacerbating airway obstruction [43].

The association between central obesity, as captured by WWI, and COPD is biologically plausible and likely multifactorial. A key putative mechanism is the systemic inflammation originating from visceral adipose tissue, which is a metabolically active organ that produces a range of pro-inflammatory cytokines [44]. This low-grade systemic inflammation, a hallmark of central obesity, can exacerbate the local inflammatory processes within the lungs, potentially accelerating the decline in lung function and contributing to the pathogenesis of COPD [45]. Furthermore, central obesity imparts a significant mechanical load on the respiratory system. The accumulation of abdominal fat can impede diaphragmatic movement, reduce lung volumes (particularly functional residual capacity and expiratory reserve volume), and increase the work of breathing [46]. This mechanical disadvantage may worsen dyspnea and limit exercise capacity, especially in individuals with pre-existing airflow limitation [47].

Our study possesses a number of noteworthy strengths. Firstly, it is grounded on nationally representative data and incorporates sample weighting, thereby enhancing the generalizability of our results to the U.S. population. Additionally, we controlled for numerous covariates in the regression analyses and utilized the extensive sample size to conduct subgroup analyses. These steps enhance the robustness and credibility of our findings.

Nevertheless, our study is subject to several limitations. First and foremost, the diagnosis of COPD relied on self-report rather than on post-bronchodilator spirometry, the established gold standard. This approach is susceptible to potential misclassification arising from recall bias or inaccurate patient understanding of the diagnosis, which could in turn affect the magnitude of the observed association. Second, although we made efforts to adjust for numerous potential covariates, the extensive array of factors that influence COPD prevents us from completely disregarding the potential influence of other unmeasured or imperfectly measured confounding factors. For instance, variables such as dietary patterns and exposure to air pollution, which are known to be associated with both obesity and COPD, were not available in our dataset. Furthermore, physical activity was defined as a dichotomous variable rather than using a more granular scale like metabolic equivalent of task (MET) minutes per week, a simplification that might not fully account for the nuanced effects of varying activity levels. Third, it is important to explicitly state that our findings demonstrate an association rather than a causal relationship. The cross-sectional nature of our research restricts our ability to infer causality. Specifically, we cannot rule out the possibility of reverse causality. It is plausible that COPD itself could lead to changes in body composition and central fat accumulation through various mechanisms, such as systemic inflammation, metabolic dysregulation, or reduced physical activity due to dyspnea, which would in turn be reflected by a higher WWI. Therefore, prospective cohort studies are required to establish the temporal sequence and causal nature of this association. Fourth, a significant number of participants were excluded due to missing data on key variables. This could introduce selection bias if the characteristics of the excluded individuals differ systematically from those included in our final analysis, potentially limiting the generalizability of our findings. However, we have adhered to the analytical guidelines provided by the NCHS by incorporating sample weights into all analyses to account for the complex sampling design and non-response, which helps to mitigate this potential bias.

## Conclusions

In conclusion, the present study has identified a noteworthy positive association between WWI and COPD, with consistent findings across various population characteristics. These findings emphasize the potential of WWI as a significant independent factor associated with the odds of having COPD. This suggests that managing central obesity, as measured by WWI, may be an important consideration in strategies aimed at mitigating the burden of COPD. Nonetheless, further basic and prospective research is required to validate these findings.

## References

[1] Sandelowsky H, Weinreich UM, Aarli BB, Sundh J, Høines K, Stratelis G, et al. COPD - do the right thing. BMC Fam Pract. 2021;22(1):244. doi: 10.1186/s12875-021-01583-w 34895164 PMC8666021

[2] Wilson N, Summers JA, Ait Ouakrim D, Hoek J, Edwards R, Blakely T. Improving on estimates of the potential relative harm to health from using modern ENDS (vaping) compared to tobacco smoking. BMC Public Health. 2021;21(1):2038. doi: 10.1186/s12889-021-12103-x 34749706 PMC8577029

[3] Nguyen BHM, Murphy PB, Yee BJ. Chronic Obstructive Pulmonary Disease and Obstructive Sleep Apnea Overlap Syndrome: An Update on the Epidemiology, Pathophysiology, and Management. Sleep Med Clin. 2024;19(3):405–17. doi: 10.1016/j.jsmc.2024.04.003 39095139

[4] Jozwiak M, Teboul J-L. Heart-Lungs interactions: the basics and clinical implications. Ann Intensive Care. 2024;14(1):122. doi: 10.1186/s13613-024-01356-5 39133379 PMC11319696

[5] Blanco I, Torres-Castro R, Barberà JA. Pulmonary vascular disease in chronic lung diseases: cause or comorbidity?. Curr Opin Pulm Med. 2024;30(5):437–43. doi: 10.1097/MCP.0000000000001091 38958570

[6] GBD 2021 Causes of Death Collaborators. Global burden of 288 causes of death and life expectancy decomposition in 204 countries and territories and 811 subnational locations, 1990-2021: a systematic analysis for the Global Burden of Disease Study 2021. Lancet Lond Engl. 2024;403: 2100–2132. doi: 10.1016/S0140-6736(24)00367-2 PMC11126520 38582094

[7] Larsson SC, Burgess S. Causal role of high body mass index in multiple chronic diseases: a systematic review and meta-analysis of Mendelian randomization studies. BMC Med. 2021;19(1):320. doi: 10.1186/s12916-021-02188-x 34906131 PMC8672504

[8] González-Gil EM, Peruchet-Noray L, Sedlmeier AM, Christakoudi S, Biessy C, Navionis A-S, et al. Association of body shape phenotypes and body fat distribution indexes with inflammatory biomarkers in the European Prospective Investigation into Cancer and Nutrition (EPIC) and UK Biobank. BMC Med. 2024;22(1):334. doi: 10.1186/s12916-024-03544-3 39148045 PMC11328449

[9] Wang H, Sui L, Xu Q, Li M, Xing Y, Li G. Common obesity-related anthropometric indices and the risk of gestational diabetes mellitus in a Chinese population: a prospective cohort study. Gynecol Endocrinol. 2024;40(1):2390848. doi: 10.1080/09513590.2024.2390848 39135447

[10] Börgeson E, Tavajoh S, Lange S, Jessen N. The challenges of assessing adiposity in a clinical setting. Nat Rev Endocrinol. 2024;20(10):615–26. doi: 10.1038/s41574-024-01012-9 39009863

[11] Sweatt K, Garvey WT, Martins C. Strengths and Limitations of BMI in the Diagnosis of Obesity: What is the Path Forward?. Curr Obes Rep. 2024;13(3):584–95. doi: 10.1007/s13679-024-00580-1 38958869 PMC11306271

[12] Tao J, Zhang Y, Tan C, Tan W. Associations between weight-adjusted waist index and fractures: a population-based study. J Orthop Surg Res. 2023;18(1):290. doi: 10.1186/s13018-023-03776-8 37038167 PMC10088134

[13] Liu H, Zhi J, Zhang C, Huang S, Ma Y, Luo D, et al. Association between Weight-Adjusted Waist Index and depressive symptoms: A nationally representative cross-sectional study from NHANES 2005 to 2018. J Affect Disord. 2024;350:49–57. doi: 10.1016/j.jad.2024.01.104 38220117

[14] Liu Y, Liang R, Lin Y, Xu B. The significance of assessing the weight-adjusted-waist index (WWI) in patients with depression: a systematic review and meta-analysis. J Affect Disord. 2025;386:119479. doi: 10.1016/j.jad.2025.119479 40419152

[15] Chen Y, Wang C, Sun Q, Ye Q, Zhou H, Qin Z, et al. Comparison of novel and traditional anthropometric indices in Eastern-China adults: which is the best indicator of the metabolically obese normal weight phenotype?. BMC Public Health. 2024;24(1):2192. doi: 10.1186/s12889-024-19638-9 39138449 PMC11321156

[16] Park MJ, Hwang SY, Kim NH, Kim SG, Choi KM, Baik SH, et al. A Novel Anthropometric Parameter, Weight-Adjusted Waist Index Represents Sarcopenic Obesity in Newly Diagnosed Type 2 Diabetes Mellitus. J Obes Metab Syndr. 2023;32(2):130–40. doi: 10.7570/jomes23005 37248034 PMC10327688

[17] Kim JY, Choi J, Vella CA, Criqui MH, Allison MA, Kim NH. Associations between Weight-Adjusted Waist Index and Abdominal Fat and Muscle Mass: Multi-Ethnic Study of Atherosclerosis. Diabetes Metab J. 2022;46(5):747–55. doi: 10.4093/dmj.2021.0294 35350091 PMC9532169

[18] Mi B, Zhang J, Jiang K, Meng H, Shan L, Hao D. Weight-adjusted waist index is a potential early predictor of degraded bone microarchitecture: A cross-sectional study of the national health and nutrition examination survey 2007-2008. J Orthop Surg (Hong Kong). 2024;32(2):10225536241268827. doi: 10.1177/10225536241268827 39075015

[19] Deng X, Wu X, Sun Z, Liu Q, Yuan G. Associations between new obesity indices and abnormal bone density in type 2 diabetes mellitus patients. Osteoporos Int. 2024;35(10):1807–15. doi: 10.1007/s00198-024-07163-9 38965122

[20] Qin Z, Chang K, Yang Q, Yu Q, Liao R, Su B. The association between weight-adjusted-waist index and increased urinary albumin excretion in adults: A population-based study. Front Nutr. 2022;9:941926. doi: 10.3389/fnut.2022.941926 36034904 PMC9412203

[21] Bianco A, Nigro E, Monaco ML, Matera MG, Scudiero O, Mazzarella G, et al. The burden of obesity in asthma and COPD: Role of adiponectin. Pulm Pharmacol Ther. 2017;43:20–5. doi: 10.1016/j.pupt.2017.01.004 28115224

[22] Kim KJ, Son S, Kim KJ, Kim SG, Kim NH. Weight-adjusted waist as an integrated index for fat, muscle and bone health in adults. J Cachexia Sarcopenia Muscle. 2023;14(5):2196–203. doi: 10.1002/jcsm.13302 37550773 PMC10570086

[23] Streba L, Popovici V, Mihai A, Mititelu M, Lupu CE, Matei M, et al. Integrative Approach to Risk Factors in Simple Chronic Obstructive Airway Diseases of the Lung or Associated with Metabolic Syndrome-Analysis and Prediction. Nutrients. 2024;16(12):1851. doi: 10.3390/nu16121851 38931206 PMC11206714

[24] Haroon S, Jordan R, Takwoingi Y, Adab P. Diagnostic accuracy of screening tests for COPD: a systematic review and meta-analysis. BMJ Open. 2015;5(10):e008133. doi: 10.1136/bmjopen-2015-008133 26450427 PMC4606431

[25] Jones PW, Harding G, Berry P, Wiklund I, Chen W-H, Kline Leidy N. Development and first validation of the COPD Assessment Test. Eur Respir J. 2009;34(3):648–54. doi: 10.1183/09031936.00102509 19720809

[26] Petersen JM, Ranker LR, Barnard-Mayers R, MacLehose RF, Fox MP. A systematic review of quantitative bias analysis applied to epidemiological research. Int J Epidemiol. 2021;50(5):1708–30. doi: 10.1093/ije/dyab061 33880532

[27] Li X, Zhao D, Wang H. Association between weight-adjusted waist index and risk of diabetes mellitus type 2 in United States adults and the predictive value of obesity indicators. BMC Public Health. 2024;24(1):2025. doi: 10.1186/s12889-024-19576-6 39075353 PMC11285432

[28] Hattab Y, Alhassan S, Balaan M, Lega M, Singh AC. Chronic Obstructive Pulmonary Disease. Crit Care Nurs Q. 2016;39(2):124–30. doi: 10.1097/CNQ.0000000000000105 26919673

[29] Cassady SJ, Reed RM. Pulmonary Hypertension in COPD: A Case Study and Review of the Literature. Medicina (Kaunas). 2019;55(8):432. doi: 10.3390/medicina55080432 31382489 PMC6723523

[30] Poh TY, Mac Aogáin M, Chan AKW, Yii ACA, Yong VFL, Tiew PY, et al. Understanding COPD-overlap syndromes. Expert Rev Respir Med. 2017;11(4):285–98. doi: 10.1080/17476348.2017.1305895 28282995

[31] Vibhuti A, Arif E, Deepak D, Singh B, Qadar Pasha MA. Correlation of oxidative status with BMI and lung function in COPD. Clin Biochem. 2007;40(13–14):958–63. doi: 10.1016/j.clinbiochem.2007.04.020 17631288

[32] Spinoza ED, Fonte FK, Carvalho VA, Dos Santos RA, Colleoni GWB, Cendoroglo MS. Body Adiposity Index as a Predictor of Body Fat in an Oldest Old and Independent Cohort of Brazilian Older Adults. Ann Geriatr Med Res. 2024;28(3):284–90. doi: 10.4235/agmr.24.0008 38757261 PMC11467517

[33] Bosello F, Vanzo A, Zaffalon C, Polinelli L, Saggin F, Bonacci E, et al. Obesity, body fat distribution and eye diseases. Eat Weight Disord. 2024;29(1):33. doi: 10.1007/s40519-024-01662-8 38710948 PMC11074037

[34] Mikolajczyk TP, Szczepaniak P, Vidler F, Maffia P, Graham GJ, Guzik TJ. Role of inflammatory chemokines in hypertension. Pharmacol Ther. 2021;223:107799. doi: 10.1016/j.pharmthera.2020.107799 33359600

[35] Holtjer JCS, Bloemsma LD, Beijers RJHCG, Cornelissen MEB, Hilvering B, Houweling L, et al. Identifying risk factors for COPD and adult-onset asthma: an umbrella review. Eur Respir Rev. 2023;32(168):230009. doi: 10.1183/16000617.0009-2023 37137510 PMC10155046

[36] Beijers RJHCG, Steiner MC, Schols AMWJ. The role of diet and nutrition in the management of COPD. Eur Respir Rev. 2023;32(168):230003. doi: 10.1183/16000617.0003-2023 37286221 PMC10245132

[37] Brock JM, Billeter A, Müller-Stich BP, Herth F. Obesity and the Lung: What We Know Today. Respiration. 2020;99(10):856–66. doi: 10.1159/000509735 33242862

[38] Wada H, Ikeda A, Maruyama K, Yamagishi K, Barnes PJ, Tanigawa T, et al. Low BMI and weight loss aggravate COPD mortality in men, findings from a large prospective cohort: the JACC study. Sci Rep. 2021;11(1):1531. doi: 10.1038/s41598-020-79860-4 33452329 PMC7810869

[39] Després J-P, Lemieux I. Abdominal obesity and metabolic syndrome. Nature. 2006;444(7121):881–7. doi: 10.1038/nature05488 17167477

[40] Mili N, Paschou SA, Goulis DG, Dimopoulos M-A, Lambrinoudaki I, Psaltopoulou T. Obesity, metabolic syndrome, and cancer: pathophysiological and therapeutic associations. Endocrine. 2021;74(3):478–97. doi: 10.1007/s12020-021-02884-x 34625915

[41] Monteiro R, Azevedo I. Chronic inflammation in obesity and the metabolic syndrome. Mediators Inflamm. 2010;2010:289645. doi: 10.1155/2010/289645 20706689 PMC2913796

[42] Saltiel AR, Olefsky JM. Inflammatory mechanisms linking obesity and metabolic disease. J Clin Invest. 2017;127(1):1–4. doi: 10.1172/JCI92035 28045402 PMC5199709

[43] Stratev V, Fjeldstad O-M. Comorbidities of COPD: Mechanisms and Treatment. Update 2023. MRAJ. 2023;11(6). doi: 10.18103/mra.v11i6.3949

[44] Wang Z, Sun Y. Unraveling the causality between chronic obstructive pulmonary disease and its common comorbidities using bidirectional Mendelian randomization. Eur J Med Res. 2024;29(1):143. doi: 10.1186/s40001-024-01686-x 38403592 PMC10895842

[45] Voynow JA, Shinbashi M. Neutrophil Elastase and Chronic Lung Disease. Biomolecules. 2021;11(8):1065. doi: 10.3390/biom11081065 34439732 PMC8394930

[46] Bagdonas E, Raudoniute J, Bruzauskaite I, Aldonyte R. Novel aspects of pathogenesis and regeneration mechanisms in COPD. Int J Chron Obstruct Pulmon Dis. 2015;10:995–1013. doi: 10.2147/COPD.S82518 26082624 PMC4459624

[47] Siddiqui S, Bachert C, Bjermer L, Buchheit KM, Castro M, Qin Y, et al. Eosinophils and tissue remodeling: Relevance to airway disease. J Allergy Clin Immunol. 2023;152(4):841–57. doi: 10.1016/j.jaci.2023.06.005 37343842

